# The experience and influence of fear after anterior cruciate ligament reconstruction: an interview study with young athletes

**DOI:** 10.1186/s13102-023-00659-7

**Published:** 2023-04-03

**Authors:** Joanna Kvist, Johanna Bengtsson, Carolina Lundqvist

**Affiliations:** 1 Unit of Physiotherapy, Department of Health, Medicine and Caring Science, Linköping University, Linköping, 581 85 Sweden; 2 Stockholm Sports Trauma Research Centre, Karolinska Institute, Stockholm, Sweden; 3 Department of Behavioural Sciences and Learning, Linköping University, Linköping, Sweden; 4 Athletics Research Center, Department of Health, Medicine and Caring Sciences, Linkoping University, Sweden

**Keywords:** Anterior cruciate ligament reconstruction, Return to sports, Fear, Psychological factors, Rehabilitation, Athletes

## Abstract

**Background:**

Despite good physical function, many athletes do not return to sports after an anterior cruciate ligament reconstruction (ACLR). One important reason for this is fear of new injury. The aim of this study was to investigate young athletes’ experiences of knee-related fear after an ACLR and how they perceive this fear to affect them in their sporting and everyday life.

**Methods:**

A qualitative interview study was conducted, using semi-structured interviews. Athletes who were active in contact or pivoting sport before an ACL injury, with the goal of returning to the same sport and who scored highly on fear of new injury at six months post-ACLR, were asked to participate. Ten athletes (six women and four men, aged 17–25 years), were interviewed by an independent researcher, 7–9 months after ACLR. Content analysis employing an abductive approach was used.

**Results:**

The analysis resulted in three categories with associated subcategories: 1. The expressions of fear; (i) reason for fear, (ii) changes in fear over time, and (iii) injury situation. 2. Reactions, consequences, and adaptations; (i) reactions, (ii) behavioural adaptation and influence on rehabilitation and daily life, (iii) present consequences, and (iv) consequences for the future. 3. Fear and adaptations related to returning to sports; (i) fear related to returning to sports and, (ii) adaptations in sports and life due to fear. Fear was described in broad and complex ways, with fear of a new injury being expressed as one of several aspects. Various reasons (e.g., seeing others getting injured in the past, previous experience of injury, failed rehabilitation, perceived knee instability) were given to explain the fear, and athletes reacted both physically and mentally to fear. Both positive and negative adaptations to fear were described, in both daily life and sports.

**Conclusion:**

The results contribute to an increased understanding of fear as an essential psychological factor to consider during rehabilitation and leaves the way open for research to investigate how physiotherapists can work to manage fear better among ACLR patients.

**Supplementary Information:**

The online version contains supplementary material available at 10.1186/s13102-023-00659-7.

## Background

Anterior cruciate ligament (ACL) injuries are common in contact and pivoting sports like football, floorball, handball, basketball and downhill skiing, with a higher incidence among elite athletes than either amateur athletes or the general population [1]. Sportswomen have a higher incidence of ACL injuries than sportsmen and injuries are 6–8 times more likely to occur during a match than during practice [2, 3]. In a study by Sonesson et al. (2016), 86% of the patients reported that returning to sport was the highest priority for the injured athlete, and most patients expect to return to their preinjury sports [4]. Returning to sport is also the main reason for having an ACL reconstruction (ACLR) [5, 6] and is often used as the primary outcome for a successful treatment after ACLR [7]. Nevertheless, not all athletes manage to return to sport. Only 55% of non-elite athletes return successfully to sport [5] compared to 83% of elite athletes [8]. Time before returning to sport varies between six and 13 months after ACLR [8]. Common criteria used for clearance to return to sport include assessment of patient reported knee function and a battery of muscle strength and hop tests. Though, a biopsychological approach has been recommended, including assessment of biological, but also psychological and social factors that may affect return to sports [9].

Psychological factors are known as common barriers to returning to sports after ACLR, despite good physical functioning [10–13], and two out of three patients report psychological factors as the reason for not returning to sports [14]. Psychological factors identified in the literature as barriers include low self-efficacy, low motivation and low readiness to return to sport, with the most important reason being high levels of fear of new injury [14, 15]. [16, 17][18, 19][20][16]Fear of re-injury after ACLR is common, and more than half of the athletes who do not return to sports report fear or not trusting the knee as their reason for not returning to sports [10, 11]. The fear of re-injury may be justified because the risk of new ACL injuries after returning to sports is high: five times higher than for athletes who do not return to sport [16, 17]. On the other hand, increased fear has a negative effect on rehabilitation delaying knee impairment resolution and readiness for advanced rehabilitation [18],[24, 25] and excessive fear of re-injury is associated with functional impairment and reduced self-reported knee function at various timepoints after ACLR [12, 18, 19]. Fear of re-injury is also described as a potential risk factor for new injuries [19, 20] that may be related to how fear affects the athlete. Fear activates the sympathetic nervous system, which influences an athlete’s physiological state, leading to: increased heart rate, hyperconscious brain, higher respiratory rate, increased blood flow to skeletal muscles and increased blood pressure [21, 22]. Autonomous functions such as cognition, memory and attention are also affected [23]. Emotional aspects of fear involve hyperactivity in the amygdala and common behavioural reactions are ‘freezing’, immobilisation followed by avoidance behaviours in relation to the frightening situation [24].

Although several studies have shown that fear affects recovery after an ACL injury [18, 25], little is known about athletes’ idiographic experiences of fear after ACLR [17]. Interview studies have revealed that fear reported after ACLR is described by participants as fear or anxiety that re-injury after returning to sports will necessitate new surgery and rehabilitation [26–28], fear of pain, being debilitated or of added financial burden [29], lack of belief in the rehabilitation process and doubts about the physiotherapist’s skills to prepare them physically and mentally for returning to sports [30, 31]. In addition, studies have demonstrated differences between men and women in their expressions of fear of re-injury; men tend to see the risk of re-injury in particular sports contexts, while women experience fear of re-injury across a broader spectrum of activities as well [32]. As fear can be perceived differently by various athletes, and can affect their rehabilitation in various ways, the aim of this study was to investigate young athletes’ experiences of knee-related fear after an ACLR and how this fear is perceived to affect them in their sporting and everyday life.

## Methods

### Study design

This study has a qualitative design with semi-structured individual interviews that were analysed with qualitative content analysis.

### Participants

Participants were recruited from an ongoing multicentre cohort study, the SPARX study, which aims to describe the return to physical activity and sports after ACLR. For the SPARX study, participants are recruited from the Swedish National ACL Registry three months after ACLR. At six months after ACLR, participants answer the ACL Return to Sport after Injury (ACL-RSI) scale to evaluate their psychological readiness to return to sport. The ACL-RSI comprises 12 questions addressing emotions, risk appraisal and confidence in performance [33]. Each question is answered on a 0–100-point Likert scale with 10-point increments, and a mean score is calculated. A higher score indicates a more positive psychological response. For the present study, we searched for eligible participants among patients who had their operations between 2 and 2020 and 14 September 2020 (a total of 111 patients). Inclusion criteria for this interview study were:

- Men and women aged 16–26 years.
- The time from primary unilateral ACLR was approximately 7–9 months, representing the time when the informants were about to return to sports and could describe their fear related to this specific rehabilitation phase.
- Active in contact or pivoting sport at least twice/week before ACL injury and reported that they wanted to return to their sport in a survey performed three months post-ACLR.
- Rated high fear (i.e. 50 or less) on two specific questions (Q) in the ACL-RSI at six-month follow-up: Q7: “Are you fearful of re-injuring your knee by playing your sport?” and Q9: “Are you afraid of accidentally injuring your knee by playing your sport?”
- Could understand and express themselves well enough in Swedish to participate in an interview.

Thirteen patients fulfilled the inclusion criteria and were contacted by mail. All participants received written information about the study and were then contacted by phone to ensure participation. Of the thirteen identified patients, one declined to participate, one was inaccessible by telephone, and one participated in the pilot interview. Thus, a total of ten patients (six women and four men, aged 17–26 years) were interviewed. The date and time for the telephone interview was determined based on each informant’s preferences. The interviews provided comprehensive and rich data that responded to the aim of the study and information from the participants began to be repeated. So, the decision was made that no more patients should be invited for interviews. Data collection was performed by the second author via qualitative semi-structured interviews during March and April 2021.

The participants were informed that the interviewer was a physiotherapist. None of the authors had any relationship with the participants. No field notes were made during or after the interviews and no interview was repeated. The transcripts were not returned to the participants for potential comments, and the participants were not asked to provide feedback on the finding.

### Interview guide and data collection

A semi-structured interview guide was designed and pilot tested before the study was conducted. This interview guide was developed by the second author, a female physiotherapist, together with the first and third authors, both female researchers experienced within sports medicine and psychology, and clinical therapists (a licenced physiotherapist/professor and a licenced psychotherapist/associate professor). The process of developing the interview guide continued until consensus was reached about the content of the questions. The semi-structured interview guide covered four main areas: (a) background information (e.g., gender, age, highest level of sports played, number of training sessions and games/week, when injury and surgery occurred), (b) experiences of fear and subjective perception of fear (e.g., “can you explain a situation, if possible during the last month, when you experienced fear in relation to your rehabilitation?”), (c) actions, thoughts and the influence of fear during the rehabilitation process (e.g., “has fear affected you in any way, positively or negatively, during the last month, for example in your rehabilitation?”), and (d) the perceived influence of fear when considering returning to sports (e.g., “do you think you will experience any fear when it is time for you to return to sports?”). The interview guide is available in Appendix 1. All interviews were conducted during the COVID-19 pandemic and therefore by phone, were audio-recorded (parts b–d) and transcribed verbatim by the second author. The first author supervised the complete data-collection process, listened to the audio recordings and checked the accuracy of the transcripts. The recorded part of the interviews lasted between 38 and 78 (mean 47.6) minutes.

### Theoretical framework and data analysis

Data was analysed inductively, underpinned by a pragmatic epistemology through a qualitative content analysis [34]. The data analysis involved four general steps: decontextualisation, recontextualisation, categorisation, and compilation of data [35]. Firstly, the transcripts were initially read and re-read by the first and second authors to gain a deep understanding of the content. Secondly, coding was performed independently by the second author to identify meaning units that captured the manifest content of the interviews. To increase dependability and ensure that the analysis process was transparent and consistent, codes and meaning units were summarised in an Excel document, together with notes about the various coding decisions made. During the coding process, and to further ensure the credibility and trustworthiness of the results, all three authors repeatedly met and discussed the relevance and content of the emerging categories, hierarchical organisation, and level of interpretation of various categories. These discussions as critical friends continued until consensus was reached between the three authors. During these discussions, the three authors also scrutinised the transcripts to ensure that no relevant data from the interviews was overlooked. Finally, the codes were organised based on their content to create sub-categories and categories to describe and understand the informants’ experiences of fear and consequences for sports and everyday life. Throughout the process, the researchers’ different perspectives and competencies (i.e., physiotherapy and psychology) continued to add depth to the analysis and the final presentation of the results. The study is reported in accordance with the Consolidated Criteria for Reporting Qualitative Research [36].

## Results

Ten patients (six women and four men, aged 17–26 years) participated in the interviews. Seven participants were active in football before their ACL injury, one in floorball, one in gymnastics, and one in parkour and climbing (Table 1).

**Table 1:** Background information of the participants

ID	Age^¶^	Sex	Sport*	Level**	Time from injury to ACLR (m)	Time from ACLR to interview (m)	ACL-RSI Q7	ACL-RSI Q9	Total ACL-RSI score
1	25	M	Football	2	2	9	30	30	52
2	25	F	Football	2	80	9	20	20	27
3	20	F	Football	1	14	9	30	20	37
4	19	M	Parkour/ climbing	3	9	8	30	30	39
5	18	M	Football	1	3	8	30	30	53
6	18	F	Football	2	8	8	30	20	43
7	17	F	Football	2	3	8	30	50	60
8	20	M	Floorball	3	49	7	10	10	17
9	26	F	Gymnastics	1	3	7	20	30	20
10	22	F	Football	1	6	7	10	10	21

M: male, F: female._ ^¶^Age at ACLR

*Sport in which the participants were active before injury and to which they want to return. **Level: 1: national elite, 2: sub-elite/competition, 3: recreational. ACL-RSI: ACL Return to Sport after Injury scale. ACL-RSI Q7: “Are you fearful of re-injuring your knee by playing your sport?”. ACL-RSI Q9: “Are you afraid of accidentally injuring your knee by playing your sport?”

**Fig. 1:**
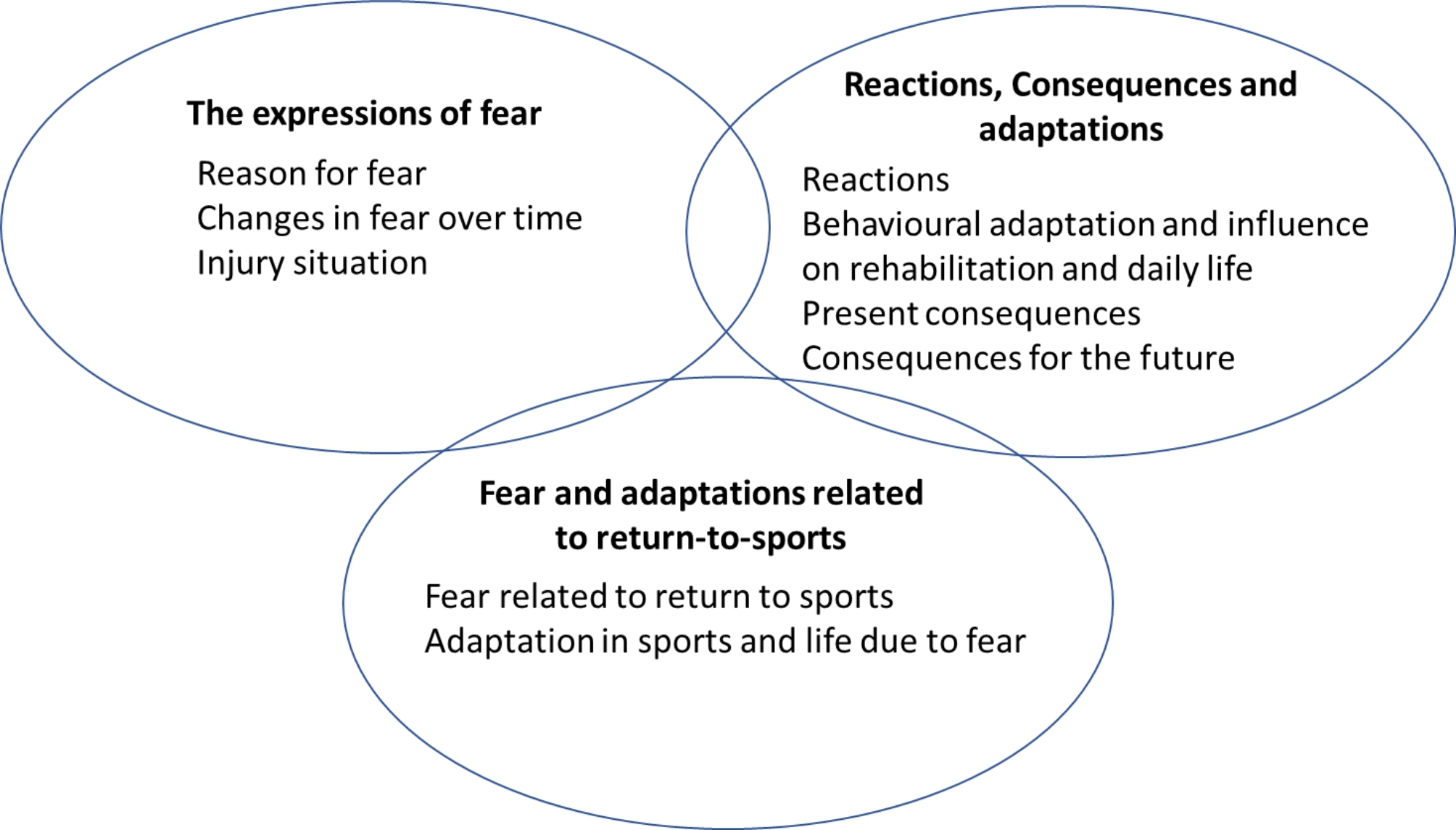
The three categories with associated subcategories describing young athletes’ experiences of knee-related fear after an ACL reconstruction and how this fear is perceived to affect them in their sporting and everyday life. Categories are interrelated to each other

The analysis of the interviews resulted in three categories, each with associated subcategories (Fig. 1). These were: (1) expressions of fear, with subcategories: reason for fear, changes in fear over time, and the injury situation; (2) reactions, consequences, and adaptations, with subcategories: reactions, behavioural adaptation, and influence on rehabilitation, and daily life, present consequences, and consequences for the future; (3) fear and adaptations related to returning to sports, with subcategories: fear related to returning to sports and adaptation in sports and everyday life due to fear.

### Expressions of fear

#### Reason for fear

For several participants, this was their first severe injury and therefore an unfamiliar situation. Being injured for the first time was experienced as scary and the fear could relate to pain, loss of control, not trusting their body and/or being limited within their sport. Having seen others getting injured in the past could increase their fear, as well as having previous experience of injury, failed rehabilitation, previous perceived knee instability, or adverse experiences of being away from sports and recreational activities.

*I had never been injured for a long time before, I just played on and then when this injury came, it [the fear] kind of came. (ID 5)*

*The feeling of [the knee] giving way is still there. And I think this is mainly what I fear will happen, even now… because it has happened so many times. (ID 2)*

Some participants described fear being initiated by the information received from caregivers when explaining about functional limitations, uncertainty about the return to sports, and the high risk of re-injury. Closely related to that, some caregivers had encouraged participants to adapt to the injury and choose other, less knee-demanding, non-contact or non-pivoting sports.

*I don’t know if that maybe…laid the foundations for my fear, but he [the doctor] said from the beginning that the risk of re-injury is very high…there are those who can’t play football any more….[so] my feeling from the beginning has been bad. (ID 10)*

The process of surgery and rehabilitation may increase experiences of fear. Participants described the rehabilitation period after the injury as psychologically challenging and time consuming. Participants also expressed fear about having to undergo a new rehabilitation period, experiencing similar pain again, and perhaps not being able to return to sports anyway. Some participants feared that a new injury would stop the progression of the rehabilitation, which would force them to start all over again.

*Then I think that [after a new injury] it’s done again, then the knee is broken, so it’s not possible to play football. So, it’s probably a fear that it won’t be good and that development progression won’t move forward. (Code 3)*

*My fear is that I will have to do more rehab [if I get injured again] and that it will … it will be longer until I can start doing what I like again. (ID 4)*

#### Changes in fear over time

Several participants described starting to feel frightened between the time from injury to surgery. For most participants, experiences of fear changed over time during the rehabilitation period. How fear was expressed varied across participants; some described fear during the entire rehabilitation period, while others mainly experienced fear related to return to sports. The fear sometimes also increased during the rehabilitation, when participants practised a new exercise or moved from one rehabilitation phase to next.

*The more I do and when I start trusting my knee, the fear fades away. So, I notice if someone pushes me, I notice yes, but I can hold on and I can lift heavy weights. (ID 6)*

Having surgery that stabilised the joint and experiencing increased functional performance reduced fear for some participants.

*It’s probably somewhat better now [after the ACLR] because then I know I have something [the reconstructed ACL], have something there that stabilises the knee again and I’m still getting better and better over time because it doesn’t sprain. (ID 9)*

#### Injury situation

The injury situation was a major factor contributing to participants’ fear, particularly if the situation was perceived as traumatic, nasty, and scary. Participants described the injury situation as frightful and something they never wanted to experience again. Several participants said that they did not fully understand what had happened when the injury suddenly occurred. A similar fear response as the one experienced in the injury situation could sometimes also arise afterwards simply from watching the sport in which they sustained their injury, during rehabilitation, or in everyday life when an exercise or situation reminded them of the injury situation. This could occur, for example, when performing a specific movement or before trying another activity or rehab exercise for the first time. Other participants described being able to perform demanding activities without fear if the activities did not resemble or remind them of the injury situation.

*I mostly relate it [the fear] to the time of the injury in that I, I’m afraid, I didn’t understand what was actually happening. Why I hurt my knee, for me it was not such a typical ‘knee injury event’. (ID 10)*

*In connection with the fact that I could start running somewhat and do high knees and such simple things, that with every step it feels as if the foot is going to get stuck in the grass again and that it’s going to be the same twist. (ID 3)*

*Because it [the injury] happened in football, when you play football, it’s like it [the fear] comes back right there and, if you do other things, you don’t think about it as much. (ID 5)*

*If you sit at home and watch football on TV… now it’s someone else who’s getting injured because now it was about the same situation. (ID 3)*

### Reactions, consequences and adaptations

#### Reactions

Physical and mental reactions to fear described by participants included the body freezing, feeling tension in the muscles, pressure over the chest, increased heart rate and uncomfortable feelings in the body. These reactions could arise, for example, before or during an activity or when exposed to slippery or uneven ground. Fear was also described by some participants as emotional reactions, such as anger or frustration. Some participants had chosen to share their fears with others, whereas some had kept these experiences within themselves and had not talked to anybody about their fear.

*I prefer not to show that I’m sad…then I show it instead by getting angry…when I get home, I go to bed and cry. (ID 10)*

*It’s probably mostly fear and that’s how you get it, you get these images back in your head of when you injured yourself. (ID 6)*

#### Behavioural adaptations and influence on rehabilitation and daily life

Participants explained various behavioural adaptations related to their fear. These could be reactive in the moment as a consequence of the sudden fear response, e.g. limping during running, or using other protective movement patterns, and thus not entirely consciously chosen. Adapting behaviours could also be a conscious choice to reduce the fear by avoiding perceived risks related to the injury, e.g. planning the foot placement during a movement, reducing their activity level or taking the safe route when walking to avoid uneven ground.

*I’m always thinking about where I put my right leg down… It’s like a reflex that now I have to think because I can’t just keep going here like you might have done if you hadn’t been injured. (ID 2)*

*If I stand still with my lower body and have to turn backwards or to the side, then I always lift my right leg which I injured … I really don’t do that with the other leg. (ID 10)*

Some participants argued that, when used as a conscious strategy, adaptions are beneficial to avoid becoming injured again or having the patience not to return to sports too soon.

*Yes, but on a positive note, it turns out that I have been, I have been more careful, which is perhaps a good thing, because then I thought a lot about what I was doing and so on. (ID 7)*

The participants’ rehabilitation was, to varying degrees, *affected* by the fear. For some participants, the fear reactions commonly became a barrier to performing an exercise, during both early and later rehabilitation and for both less and more demanding exercises. It was perceived as difficult to fully involve the injured leg in exercises and, for example, jumping from a low height could make them terrified of pain. Several participants said that they were more cautious during their rehabilitation to protect the knee.

*We would start jumping and do different jumping tests and things like that. Then it [the fear] has hindered me, as if I simply didn’t dare. (ID 7)*

*I’m a bit careful with weights, for example… I don’t put on a lot when I do, for example, squats with a barbell…it’s probably mostly because I’m afraid. (ID 6)*

Other participants argued that the forces to which the knee joint is exposed during sports cannot be achieved by doing rehabilitation exercises. Several participants expressed a need for physical support to help them increase their confidence in doing simple tasks.

*I had to hold his [the physiotherapist’s] hand and it was like, I screamed out of fear when I was going to jump up, and then I jumped 10 cm. (ID 2)*

Adaptations sometimes led participants to avoid feared situations that could be beneficial for their rehabilitation in the long run. These avoidance behaviours were expressed, for example, by interrupting an exercise or physically moving away from the situation. Thus, some adaptions and strategies used by participants to manage fear negatively influenced or delayed their progression in rehabilitation. Although fear made participants adjust how they performed an exercise, they did not always avoid doing the exercise completely. Some participants managed to continue exercises despite their fear by increasing focus on being more careful, being overly protective of the knee, or being closely observant of their actions, pain and fear. If the participants’ attention became too focused on the knee, instead of the rehabilitation, the fear was perceived as a distractor during training. For example, some participants explained that they were highly focused on how to land after a jump, relating to the foot-knee-hip position, instead of focusing on the power and performance of the jump.

*Then maybe it would have gone even easier if I hadn’t had it [fear], but it’s not like I’m giving up certain things in the rehab or anything like that, but we’re moving on. (ID 2)*

*I think more about my knee landing straight ahead, i.e. in line with my foot, than breathing, for example. (Code 9)*

Some participants explained that being exposed to frightening and demanding exercises sometimes helped them to reduce fear over time, but that it had been difficult to overcome fear related to some exercises or activities, for example activities similar to the injury situation.

#### Present consequences

Experiences of how the fear affected everyday life and leisure time varied across participants and affected everyday life to a greater or lesser extent. Fear could lead participants to adjust to a slower pace when walking downhill, and it also affected their social lives; for example, with less engagement in recreational sports that had been a part of their everyday life before the injury. Some participants also stated that the fear, in combination with their functional limitations, affected their work.

… *if it’s quite a steep downhill, I have a much slower pace than I would have if I hadn’t had that fear, sort of. (ID 2)*

*We often do a lot of active things in our free time… I can’t seem to do everything I want to do… like go away and play padel. (ID 6)*

*At work or on a normal day, if I lift something and I …go backwards while carrying something quite heavy … then I feel … discomfort in my whole body. (ID 9)*

#### Consequences for the future

Participants mentioned both concerns and positive consequences for the future related to the injury and fear. Concerns related to doubts about being able to address future knee problems without fear, the timespan during which they would be reminded of the injured knee, having difficulties overcoming the injury situation and being restricted physically in the future if the rehabilitation was not properly carried out. Some were also afraid of sustaining new, more serious injuries in the future.

*I think about it all the time… I would say that I think about my knee once an hour in my everyday life… linked to fear and limitations, not such positive thoughts. (ID 2)*

*I want to make sure I’m able to, yes, but like playing floorball, skiing, going out and running without thinking, thinking about every single step I take. (ID 9)*

Some participants described a desire to improve their skills in dealing with adversity to decrease future fear. Several participants also said that the injury, surgery and rehabilitation had increased their awareness and respect for knee function. The injury experience also provided some positive consequences in terms of increased resilience and lessons learned that made participants grow as people.

*I have so much more respect… There can be things that happen that mean you can’t use your body the way you want and that, I really hadn’t thought about that before, before I injured myself. (ID 4)*

*I have to develop as a person related to this circumstance because I need to learn how to handle certain situations and deal with adversity. (ID 10)*

### Fear and adaptations related to returning to sports

#### Fear related to returning to sports

Some participants predicted that the fear would be so intense that it would prevent them from returning to sports, or they feared returning to sports prematurely and therefore becoming re-injured. Other participants did not expect football training to be problematic but rather anticipated that fear during matches could be obstructive in certain situations during the match or sports environments that resembled the injury situation. Participants also described fears related to opponents being unaware of their injury and not being as careful as their teammates.

*I don’t know, but it’s clear that, if I choose not to play matches, it’s actually because of fear, fear of getting injured again. So it’s clear that it [the fear] may well arise. (ID 1)*

*Because they [the opponents] don’t know what I’ve been through, while the team knows, yes, what I’ve been through and how much I rehabbed and stuff like that… that I’ve been injured. (ID 6)*

#### Adaptations in sport and life due to fear

Among participants who aimed to return to the same sport, different behavioural adaptations were common. These adaptations included returning to a lower level as a first step; for example, to start playing in a lower division or getting back to the sport step by step. In addition, some were thinking about changing position within the team or turning to exercise or playing another sport instead of football. Some participants perceived alternative activities other than football as easier to return to, despite being similarly demanding on the knee.

*I’ve always wanted to get back to Division 1 level anyway. But we’ll see if it will be Division 1 level at once, or if I’ll play for a team in a lower division first. (ID 3)*

*I’ve probably thought about retraining myself positionally… I think that as an outside back I’m less exposed than I am as an inside midfielder. … it’s clear that I’m exposed as an outside back too, but … I have a little more control then. (ID 10)*

*It feels so contradictory that I can come back to padel but not football…, it could simply be about my fear of football… I don’t have the same feeling of anxiety about padel as I have about football. (ID10)*

## Discussion

Fear related to sports injuries has mostly been described as fear of new injury [17]. In the present study, however, fear was described by our participants in broader and more complex terms. The results revealed that participants’ previous experiences, the traumatic injury situation, their contact with healthcare services (information and treatment) and the risk of new injury were all reasons for fear. Participants responded to fear both physically and mentally, and were affected by fear during rehabilitation, sports and daily life, which also led them to adopt adaptations in different aspects of life. In line with the concept of sports-injury-related growth, defined as *“perceived changes that propel injured athletes to a higher level of functioning than that which existed before their injury”* (p.36) [37], several participants in this study described how the injury and rehabilitation had provided new insights that helped them in their personal development. Finally, participants explained how fear affected their return to sports and the adaptations they found it necessary to make in relation to that return.

The length of time between the injury and ACLR and participants’ perceived knee instability before the ACLR were related to fear. Only four of our participants had an early ACLR, within three months of the injury. As our participants were active in contact or pivoting sports before the ACLR, early reconstruction would have been expected [6, 7]. Prior research has reported similar results, showing that patients who wait more than three months between injury and ACLR have a greater risk of fear [38]. Patients who go for late ACLR make that decision due to functional problems with the knee, feelings of instability and episodes of giving way [6], and these patients report greater fear three months after injury than those who decide not to have an ACLR [39]. For the clinician, it can be a dilemma to decide if an early ACLR should be performed before the fear ‘gets the athlete’, while also being aware of the increased risk of re-injury with early ACLR and a rapid return to sport [16], and that not being psychologically prepared for the long rehabilitation may also have a negative effect on the athlete’s treatment outcome [10].

A significant reason for participants’ fear was the information received from caregivers about functional impairments, the low rates of athletes returning to sports and the increased risk of new injuries. It is essential that health professionals inform the patient about the injury and its prognosis so the patient can make informed decisions both about the treatment [40, 41] and about their return to sports [9]. Presenting a picture that is too optimistic about the situation after an ACLR can decrease treatment satisfaction [42], but being too pessimistic can reduce motivation during the rehabilitation [4] and induce fear. Nonetheless, healthcare professionals should make an effort during the initial contact with the patient to carefully consider how information can best be communicated to ensure the patient is well-informed [40, 41], while also following up how the patient understands the information in order to avoid unnecessary fear or misunderstandings about the rehabilitation or prognosis.

Participants in this study described both reactive and conscious behavioural adaptations as common ways to reduce fear or avoid perceived risks related to the injury. Although some participants interpreted these reactions and adaptations – such as hyperfocus on the knee, freezing and stiffening the body or trying to control the body in threatening situations – as being positive ways to reduce their short-term fear, these behaviours may have a negative effect on rehabilitation and preparation for the return to sports. Freezing and stiffening the body has been demonstrated to increase EMG activity and muscle co-activation during landing in patients with high levels of fear [43] and as an adaptation strategy in patients with poor knee function [44]. Fear is related to the adoption of safety behaviours, i.e., actions or subtle cognitive strategies (e.g., escape, avoidance, distraction or use of other safety cues). Safety behaviours are commonly used prior to confrontation with the intention of reducing momentary discomfort, feel safer or reduce harm when actually confronted with an anticipated feared stimulus. Although reducing momentary fear, safety behaviours can also act to maintain future fear by preserving threat beliefs and prohibiting new learning [45, 46]. Hyperfocus on the knee and hyperawareness have been identified as an umbrella theme in relation to fear in previous research [27]. Hyperawareness increases the internal focus during rehabilitation, leading the athlete to think more about protection of the injured body part (e.g. knee-over-toes position), instead of the preferred external focus, such as kicking a ball or jumping to reach a ball [47]. Participation in sports demands full attention to the play or the match (e.g. the ball, the opponent, one’s teammates) and fear reactions reduce the attention paid to necessary aspects of the skills execution, and thus can be a potential risk factor for new injuries [19, 20].

Fear was not constant among participants, but changed during their rehabilitation, as has been described previously [18, 39]. Important timepoints affecting fear were the surgery, progression between different phases during rehabilitation and when the participants managed to perform (and overcome fear) during specific rehabilitation tasks. High levels of fear during the initial stages of rehabilitation affect both the progression of the rehabilitation and the outcome [18]. Participants described gaining confidence as they attempted and then managed to perform new tasks (afraid at first, but then better). Today, systematic exposure in cognitive-behavioural therapy (CBT), which is based on principles of fear extension, is the primary evidence-based method for the treatment of anxiety disorders and fear, and has extensive research support [46, 48–50]. Exposure is a set of therapeutic psychosocial strategies in which the patient systematically and repeatedly approaches fear-provoking stimuli (e.g., objects, situations, interoceptive cues, memories), rather than avoiding them, without the feared outcome occurring. Thus, during rehabilitation, this technique would be employed to support injured athletes in exposing themselves to feared exercises. Preferably, exposure would be performed with exercises that resemble the execution of skills with which they will be confronted when returning to sports, instead of avoiding or overprotecting the injured body part because of fear. The goal of exposure therapy is therefore to reduce fear and anxiety about objectively non-harmful stimuli. Fear habituation, belief disconfirmation and inhibitory learning are proposed as mechanisms, with cognitive factors and new learning suggested to play a major role. Exposure provides new learning about the association between the feared stimulus and the actual outcomes, resulting in fear reduction, although research indicates that associations learned during fear conditioning are not erased, but provide secondary inhibitory learning [48, 49].

Our participants were 7–9 months after ACLR, i.e. during the final phase of rehabilitation, targeting their return to sports. They described feeling confident in doing the rehab exercises because they knew that the force on the knee was much less than in a match situation. The extinction of fear seemed, however, to be context specific, and fear might therefore return when the threatening stimulus appeared in a different context than the one in which exposure took place [15, 24]. This suggests that injured athletes should be exposed to exercises they fear in various settings, and not only in the clinic. Participants also described increased fear and insecurity about going back to the same sports in which they were injured and fear about meeting opponents who may not know that they have been injured. During this phase of rehabilitation, on-field rehabilitation [51] plays an important role in increasing functional performance and enabling the patient to exercise and be exposed to real situations in order to gain confidence.

Our participants also described feelings of anger and frustration that may shift their focus away from the rehabilitation. Emotions are known to potentially have an impact on attention and performance by limiting the information-processing capacity and leading the athlete to focus on irrelevant information instead of the information needed for proper task execution [52]. Thus, supporting the athlete with functional emotional regulation strategies and encouraging them to maintain attention on the task-execution of rehabilitation exercises might be valuable. Many participants described how the fear affected them in everyday life. Some did not show or discuss their fear with others, maybe due to the stigmatisation of mental health services [53]. Fear also caused them to avoid social situations that may be demanding on the knee. The importance of social support has previously been highlighted and can result in more athletes managing to return to sports [29, 53].

This study presents several interesting findings and transferability to other populations of athletes after ACL-reconstruction was facilitated through the clear description of patient recruitment with narrow inclusion criteria for the participants. All participants were between seven and nine months after ACLR, wanted to return to contact or pivoting sports and had reported high fear at six months postoperatively. The inductive approach of this study also fits well with the study aim to describe and understand the participants experiences of fear and consequences fear might have for return to sports and general life. One strength of this study is the multi-professional research team, which included both sports physiotherapist and sports psychologist. Nevertheless, this study is not without limitations. The results are obtained from a small sample of ACL-injured Swedish athletes from national elite to recreational level represented predominantly by football players having surgery and rehabilitation in different places in Sweden. As a result, the participants experience of fear may differ from other athletes suffering other injuries, playing other sports or being from other countries or cultures. Thus, the results should be interpreted with an appropriate contextual and cultural framework. Moreover, data collection was performed during the COVID-19 pandemic. Sweden was an open society with limited social distancing restrictions during the pandemic, but stressors and worries experienced in the general population during this period might still have altered participants experiences of fear. When interpreting the results, readers should therefore keep in mind that the pandemic were ongoing, and that circumstances were extraordinary at that period of time.

## Conclusion

Fear was described in a wide and complex range of ways, with fear of a new injury described as one of several aspects of fear. Various reasons (e.g., seeing others being injured in the past, previous experience of injury, failed rehabilitation, perceived knee instability) explained the fear, and athletes reacted both physically and mentally to their fear. Both positive and negative adaptations to fear were described, both in daily life and while playing sports. The results contribute to an increased understanding of fear as an essential psychological factor that must be considered during rehabilitation and leave the way open for research to investigate how physiotherapists can work to better manage fear among ACLR patients.

## Electronic supplementary material

Below is the link to the electronic supplementary material.

Supplementary Material 1

## Data Availability

Deidentified data is available from the first author, Joanna Kvist (joanna.kvist@liu.se ) upon reasonable request.
