# Supplementary material for: The experience and influence of fear after anterior cruciate ligament reconstruction: an interview study with young athletes
Source: BMC Sports Sci Med Rehabil. 2023 Apr 3;15:50. doi: 10.1186/s13102-023-00659-7 (PMC10068218; doi:10.1186/s13102-023-00659-7)
Supplement: Supplementary file 1 — Supplementary Material 1 [file 13102_2023_659_MOESM1_ESM.docx]

**The experience and influence of fear after anterior cruciate ligament reconstruction: an interview study with young athletes**

**INTERVIEW GUIDE**

**Background information**

Gender, age, sport, highest level, number of training sessions/matches per week, occasion of injury and surgery.

**Introduction**

-How is your rehabilitation going?

-How far have you come in your rehabilitation?

- Have you encountered any complications or setbacks so far?

The reason you are participating in this interview study is because you have reported fear of new injury at your six-month follow-up in the other study you are participating in.

1. Can you tell me how you experience this fear?

2. Can you tell me about any situation in which you experienced fear in connection with your rehab?

- Regarding X, can you imagine what happened in that situation?

- How did you react physically and mentally in that situation?

- Do you have more examples?

3. Can you tell me if there were other situations outside of rehab and sports when you experienced this fear? (Last month)?

4. Have you ever experienced similar fear when only thinking about a certain situation?

5. Has the fear affected you in any way, positively or negatively (last month)?

- Examples in rehab

- Examples in other contexts

6. Can you tell me if your experience of that fear has prevented you from doing something? Last month?

- Examples in rehab/sports

- Examples outside rehab/sports

7. How do you act in situations where you experience fear?

- Physical reactions

- Mental reactions

8. Can you tell me if you have done anything to deal with your fear?

Own strategies (coping, methods, avoidance)

Have you received support/help? (Other people around, support, physio)

9. Can you tell me how you see your return to sports?

What does the plan look like for your return to sports? (When? Do you think you will return? To the same sport?)

Do you feel that there are any obstacles for you to return to your sport?

How do you see your life in six months? What do you think you will be doing then?

10. When the time arrives to return to sports, do you think you might experience fear then?

Have you ever felt fear/worry about experiencing fear in the future?

11. Would you like to add anything that you think is important to this topic?

Finally, the informants are allowed to summarise.

Exit. Connect, thank them for their participation.

Follow-up questions:

What you said about X was interesting, can you tell me more about X?

Can you imagine yourself in that situation again?

How did you react to X?

What were you thinking when X happened?

What happened in that situation?

What do you mean by X?

Can you give more examples?
